# The reduction of miR146b-5p in monocytes and T cells could contribute to the immunopathogenesis of hepatitis C virus infection

**DOI:** 10.1038/s41598-019-49706-9

**Published:** 2019-09-16

**Authors:** Yasuteru Kondo, Takayuki Kogure, Masashi Ninomiya, Ryo Fukuda, Norikazu Monma, Kazuho Ikeo, Yasuhito Tanaka

**Affiliations:** 1 Department of Hepatology, Sendai Kousei Hospital, 4-15 Hirose, Aoba, Sendai City, Miyagi Japan; 2 Division of Gastroenterology, Tohoku University Hospital, 1-1 Seiryo, Aoba, Sendai City, Miyagi Japan; 3 Center for information Biology, National Institute of Genetics, Mishima, Japan; 4 Department of Virology & Liver unit, Nagoya City University Graduate School of Medical Sciences, Kawasumi, Mizuho, Nagoya 467-8601 Japan

**Keywords:** Hepatitis C, Hepatocellular carcinoma

## Abstract

It has been reported that various kinds of miRNAs could affect the pathogenesis of hepatitis C virus infection. Recently, our group reported that deep-sequencing analysis was useful to detect disease-specific miRNAs. The aim of this study is to identify the HCV-specific miRNAs that could contribute to the immunopathogenesis of HCV by using clinical samples and *in vitro* analysis. Five miRNAs (hsa-miR181a-2-3p, hsa-miR-374a-3p, hsa-miR374a-5p, hsa-miR-204-5p and hsa-miR146b-5p) were shown to be significantly downregulated in CH-C by deep sequence analysis. The average ratio (PBMCs miRNAs/serum miRNAs) of hsa-miR146b-5p was highest among all the miRNAs. Moreover, serum hsa-miR146b-5p was significantly down-regulated in CH-C patients in comparison to CH-B patients and healthy subjects. The expression of hsa-miR146b-5p in CD3^+^ T cells and CD14^+^ monocytes of CH-C patients was significantly lower than that of the other groups. The hsa-miR146b-5p expression in CD14^+^ monocytes of SVR patients treated with Peg-IFN/RBV was significantly higher than in those of non-SVR patients treated with Peg IFN/RBV. However, the hsa-miR146b-5p expression in CD14^+^ monocytes of SVR patients treated with DCV and ASV was comparable to that in monocytes of non-SVR patients treated with DCV and ASV. Moreover, the expression levels of hsa-miR146b-5p in CD14^+^ monocytes were significantly increased after achieving SVR and 1(OH)Vitamin D3 treatment. Further, the expression of HCV-Core could suppress miR146b-5p expression in immune cells and affect the expression of various kinds of cytokines by affecting the NF-κB signaling. In conclusion, the reduction of miR146b-5p in monocytes and T cells could contribute to the immunopathogenesis of hepatitis C virus infection.

## Introduction

Hepatitis C virus (HCV) is a non-cytopathic RNA virus that causes persistent inflammation, fibrosis and hepatocellular carcinoma (HCC)^1^. HCV has multiple strategies to evade host immunity that include innate immune systems and adaptive immune systems^2^. Although the use of direct antiviral agents (DAAs) has resulted in a significant improvement in the rate of sustained virological response (SVR), new issues including viral mutation, relapse, re-infection, appearances of HCC after SVR and the remarkably high cost of DAAs therapy should be considered^3–11^. Moreover, the lack of an effective vaccine for HCV is one of the important problems in controlling this global infection. Better understanding of the host immunity to HCV might solve these issues.

In addition to innate immune systems, adaptive immune systems have an important role in the immunopathogenesis of HCV infection^12–15^. HCV persistent infection causes a dysfunction of cellular immune responses including those of dendritic cells (DCs), type 1 helper T cells (Th1 cells) and cytotoxic T lymphocytes (CTLs) etc. On the other hand, the excessive function of the immune suppressive cells including regulatory T cells (Tregs) and myeloid-derived suppressor cells (MDSCs) could contribute to the persistent infection of HCV, Hepatitis B virus (HBV) and the pathogenesis of difficult-to-treat chronic hepatitis C (CH-C) and hepatocellur carcinoma (HCC)^16,17^. Moreover, the dysfunction of monocytes could also contribute to the immunopathogenesis of CH-C. The disturbance of the immune system in CH-C patients might occur by the indirect and direct effects of HCV^13,18–23^. Many groups including ours reported that HCV infection could induce autoimmune-related diseases including rheumatoid arthritis and cryoglobulinemia, etc.^12,24^. Previously, we reported that the administration of 1(OH)vitamin D3 could stabilize the excessive production of interferon γ- inducible protein 10 (IP10) from monocytes^25^.

Micro RNAs (miRNAs) are small endogenous noncoding RNAs that pair with target messengers regulating gene expression. They bind to complementary sequences in 3′ UTR of target messenger RNA (mRNA) transcripts, usually resulting in translational repression, the inhibition of protein synthesis, and gene silencing^26–31^. It has been reported that crucial immune functions could be regulated by specific miRNAs^32^. Various kinds of miRNA including miR146, miR155, miR181b, miR-21 and miR301a were reported to affect NF-κB signaling^33^. miR146 is an NFκ-B transactivational target that negatively regulates IRAK1 and TRAF6, constituting a negative feedback loop^33^. It has been reported that a decrease of miR146b-5p in monocytes is associated with loss of the anti-inflammatory action^34^.

In this report, we identified specific miRNAs that were significantly downregulated in CH-C patients compared to chronic hepatitis B (CH-B) patients and healthy carriers by using deep sequencing analysis. Moreover, we identified the biological significance of miRNA146b-5p, which was significantly suppressed in CH-C. Moreover, the addition of 1(OH) vitamin D3 could recover the expression of miRNA146-5p.

## Materials and Methods

### Study design and patients

**Figure 1:**
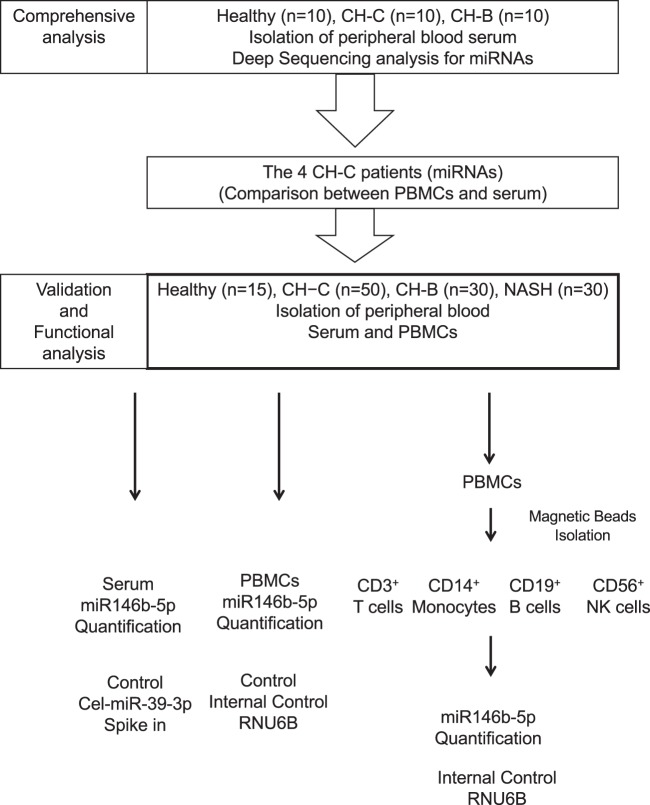
The scheme of this study is shown in this figure. A comprehensive, validation and functional analysis was carried out in this study.

This study was approved by the Ethics Committee of Tohoku University School of Medicine (2013-1-268) following ethical guidelines of the 1975 Declaration of Helsinki, and written informed consent was obtained form each individual. We included the serum of 10 treatment-naïve CH-B patients, serum of 10 treatment-naïve patients with HCV chronic infection (CH-C) and the serum of 10 healthy controls to carry out deep sequencing analysis. The diagnosis of all cases enrolled in this study was based on internationally established criteria. For the replication study, we included 30 treatment-naïve CH-B patients, 30 treatment-naïve non-alcoholic steatohepatitis (NASH), 50 treatment-naïve CH-C patients and 15 healthy controls (Fig. 1). The diagnosis of all cases enrolled in this study was based on internationally established criteria. These procedures were carried out in accordance with the approved guidelines.

### Library preparation and Illumina sequencing

For each participant, a 10 ml venous sample was collected and separated into serum by centrifugation at 2,500 rpm for 10 min. Samples were stored at −20 °C until further analysis. Total RNA was extracted using Trizol LS (Invitrogen, Carlsbad, CA). Moreover, total RNA was extracted from PBMCs of 4 selected CH-C patients. The libraries were constructed as previously described using the TruSeq Small RNA Sample Prep Kit. Libraries were sequenced on illumina GC IIx (SCS 2.8 software; Illumina, SanDiego, CA) with a 32-mer single end sequence. Image analysis and base calling were conducted using RTA 1.8 software^35^.

### Sequence and statistical analysis

**Figure 2:**
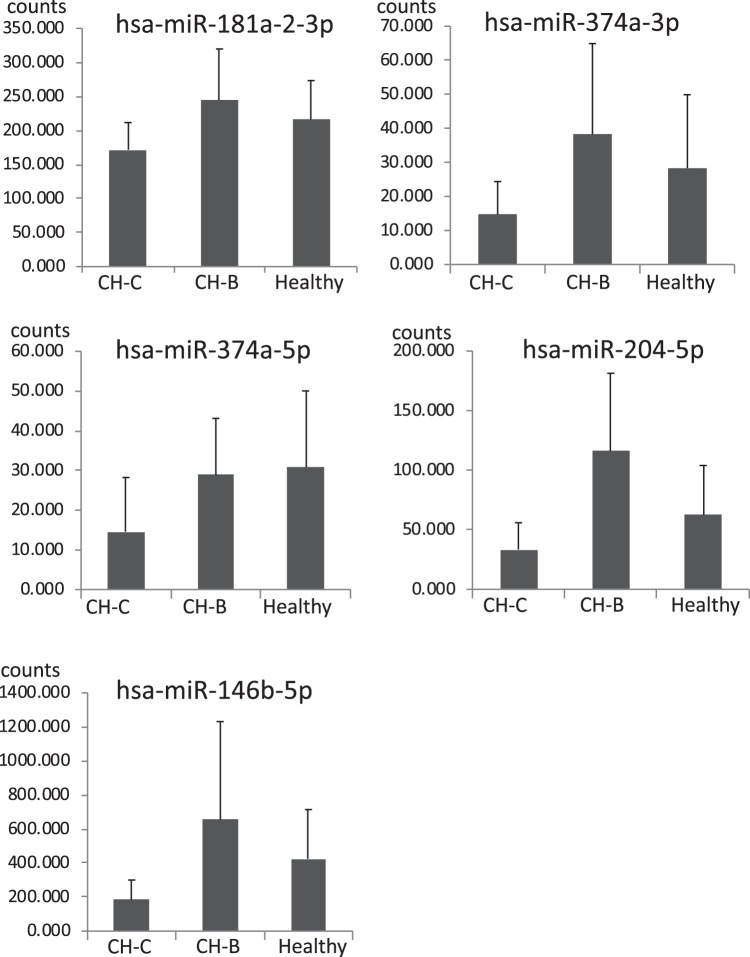
The CH-C specific suppression of miRNAs in the serum. This figure shows the five miRNAs that had significantly lower expression in CH-C patients in comparison to those in the other groups. Y-axis indicates the counts of miRNAs. Error-bars indicate standard deviation.

A quality check was performed on raw miRNA sequence reads and the adapter sequences were removed by cutadapt. The sequence reads were aligned with miRBase (Release 18) and UCSC (hg19) by use of bwa (0.5.9-r16), allowing one nucleotide base mismatch. The trimmed mean of the M value (TMM) normalization method was applied to these RNA-sequence data by calculating the digital expression levels of the counts of miRNAs. Read counts of each detected miRNA were normalized to the total number of miRNA reads, and the ratio was then multiplied by a constant set to 1 × 10^6^ in this study. The extracted differentially expressed miRNAs among the three groups were processed by ANOVA. The *p*-values of multiple comparisons were performed by calculating the FDR. Those miRNAs with FDR < 0.1 were extracted as differentially expressed^35^ (Fig. 2).

### Isolation of PBMC, various kinds of immune cells and flow cytometry analysis

PBMCs were isolated from fresh heparinized blood by means of Ficoll-Hypaque density gradient centrifugation (Amersham Bioscience, Uppsala, Sweden). Isolation of CD3^+^ T cells, CD14^+^ monocytes and CD19^+^ B cells was carried out using a magnetic beads method. The purity of isolated cells was analyzed by FACS canto-II (BD, San Jose, CA). The FACS data were analyzed by FlowJo 7.6 software.

### Quantification of cytokines in the culture supernatant

The amounts of CXCL10, TGF-β1, IL10, IFN-α, IL12, TNF-α and IFN-γ were quantified using enzyme-linked immunosorbent assay (ELISA) kits (RayBiotech, GA)(PBL Assay Science, NJ)(R &D Systems, MN). The culture supernatants were collected at 72 hours post miRNAs transfection and stored at −20 °C. The ELISA procedure was performed according to the manufacturer’s protocol.

### Realtime-PCR validation study

Fifty serum and various immune cell (PBMCs, CD3^+^ T cells, CD14^+^ monocyte, CD19^+^ B cells and CD56^+^ NK cells) -samples of CH-C, 30 samples of CH-B, 30 samples of NASH, 15 samples of healthy carriers were used in a real-time PCR validation study. We followed the protocol previously established to determine the endogenous miRNA levels with spiked-in Caenorhabditis elegans miRNA-39 (cel-miR-39)(5′-UCA CCG GGU GUA AAU CAG CUU-3′). Realtime PCR analysis was carried out to detect hsa-miR146b-5p using a TaqMan MicroRNA assay with StepOne Plus detection system (Applied Biosystems). The data were analyzed by the 2^−ΔΔCt^ methods.

### Transfection of HCV individual protein expression plasmids

Various expression plasmids were constructed by inserting HCV-core, E1, E2, NS3, NS4B, NS5A and NS5B cDNA of genotype 1a behind the cytomegalovirus immediate-early promoter in pCDNA3.1 (Invitrogen). Primary CD4^+^ cells were transfected using 4D-Nucleofector II (Amaxa, Gaithersburg, Washington DC, USA) with a human T cell nucleofector kit (Amaxa), and the various plasmids were purified using the EndFree plasmid kit (QIAGEN, Valencia, CA, USA). Viable transfected cells were isolated by Ficoll-Paque centrifugation (Amersham Bioscience) at 24 hours post-transfection. The transfection and expression efficiencies were analyzed using intracellular staining of individual proteins of HCV and flow cytometry analysis^12^.

#### Cell culture derived JFH-1 clone

Cell-culture-derived, infectious HCV was generated as described previously^36^. The HCV was quantified as follows: RNA was extracted from the Huh7 culture supernatant using the QIAamp Viral RNA Kit (Qiagen, Valencia, CA). The HCV RNA was quantified by real-time reverse transcription polymerase chain reaction using TaqMan EZ RT-PCR Core Reagents (Applied Biosystems, Foster City, CA) according to the manufacturer’s protocol using the published primers and probe^37^. The filtered (0.45 µm) culture supernatant of HCV-infected Huh-7 cells containing 2 × 10^8^ HCV RNA copies/mL (equivalent to 9.7 × 10^4^ focus-forming units [ffu]/mL) was used for the experiments^38^.

### Cell culture and transfection of miRNA

A human monocytic cell line (THP-1 cells) and T cell line (Jurkat cells) were used for the analysis of miRNA inhibitor or mimic transfection. The THP-1 cell line and Jurkat cell line were cultured in serum-free medium. The inhibitor and mimic of hsa-miR146b-5p were purchased from Applied Biosystems. The transfection of miRNA was carried out using 4D-Nucleofector (Lonza, Walkersville, MD, USA). The total RNA was isolated from THP-1 cells and Jurkat cells to carry out real-time PCR at 48 hours post-transfection.

### Network analysis

To identify the potential roles of miR-146b, protein-to-protein interaction of the target genes of miR-146b were analyzed. DIANA-TarBase v7.0, a database that indexes experimentally validated microRNA targets, was used to identify the target genes of miR146b. The list of target genes is shown in Supplemental Table 3. The gene functional classification tool from the Database for Annotation, Visualization and Integrated Discovery (DAVID) was used to identify enriched gene ontology (GO) terms in the target genes of miR-146b. The protein-to-protein networks of the target genes were analyzed using the String v10 program.

### NF-kB transcription assay

THP-1 cells were electroporated with 300 nM of miR-146b mimic or negative control (Ambion Life Technologies, Carlsbad, CA, USA) using P3 Primary Cell 4D-Nucleofector™ X Kit (Lonza, Walkersville, MD, USA). After 48 hours of transfection, cells were treated with TNF-α (20 ng/ml, Wako, Japan) or diluent control for 6 hours. The nuclear extract from the cells was prepared using a TransAM Nuclear Extract Kit (Active Motif, Carlsbad, CA) according to the manufacturer’s instructions. Briefly, cells were resuspended with hypotonic buffer for 15 min on ice to swell the cells. Cells were vortexed with detergent and centrifuged at 14,000 g for 30 sec at 4C. The nuclear pellets were resuspended in lysis buffer with detergent and incubated on ice for 30 min. The lysates were centrifuged at 14,000 g for 10 min and the concentration of nuclear extracts was determined using a Bradford assay kit (Pierce, Rockford, IL, USA). The transcription activity of NFκ-B was measured using an NF-κB p65 transcription factor assay kit (Active Motif). Briefly, 20 ng of nuclear extract were diluted with complete binding buffer and added to wells of a 96-well plate coated with oligonucleotide containing NF-κB consensus site 5′-GGGACTTTCC-3′, and the plate was incubated at room temperature for one hour. NF-κB antibody was added to each well and incubated at room temperature for one hour. HRP-conjugated secondary antibody was added and incubated for one hour. Developing solution was added and the absorbance of each well was measured using a spectrophotometer at 450 nm wave length.

### Reporter assay

Reporter assay was conducted using miRNA 3′ UTR target clones with firefly luciferase gene as the miRNA 3′UTR target reporter and Renilla luciferase gene as the internal control. Immune cells (THP-1 and Jurkat) or HCC cells (PLC/PRF/5) were electroporated with pEZX-MT06 target reporter vectors containing 3′ UTR of genes (GeneCopoeia, Rockville, MD, USA) together with miR-146b mimic or negative control (Ambion Life Technologies, Carlsbad, CA, USA) using a 4D-Nucleofector (Lonza, Basel, Switzerland). After 24 hours, the cells were collected and luciferase assay was performed using Luc-Pair Luciferase Assay Kit 2.0 (GeneCopoeia) according to manufacturer’s instructions. Luciferase activity was measured using Varioskan™ LUX multimode microplate reader (Thermo Scientific, Madison, WI, USA). Luminescence of the firefly luciferase was normalized using the Renilla luciferase. Relative luminescence of a reporter vector co-transfected with miR-146b mimic was calculated as the ratio for the negative control. pEZX-MT06 target reporter vectors containing 3′ UTR of genes (HmiT054504-MT06 for NFKB1, HmiT017891-MT06 for MAP3K7 and HmiT018237-MT06 for TRAF6) were purchased from GeneCopoeia.

### Statistics

**Figure 3:**
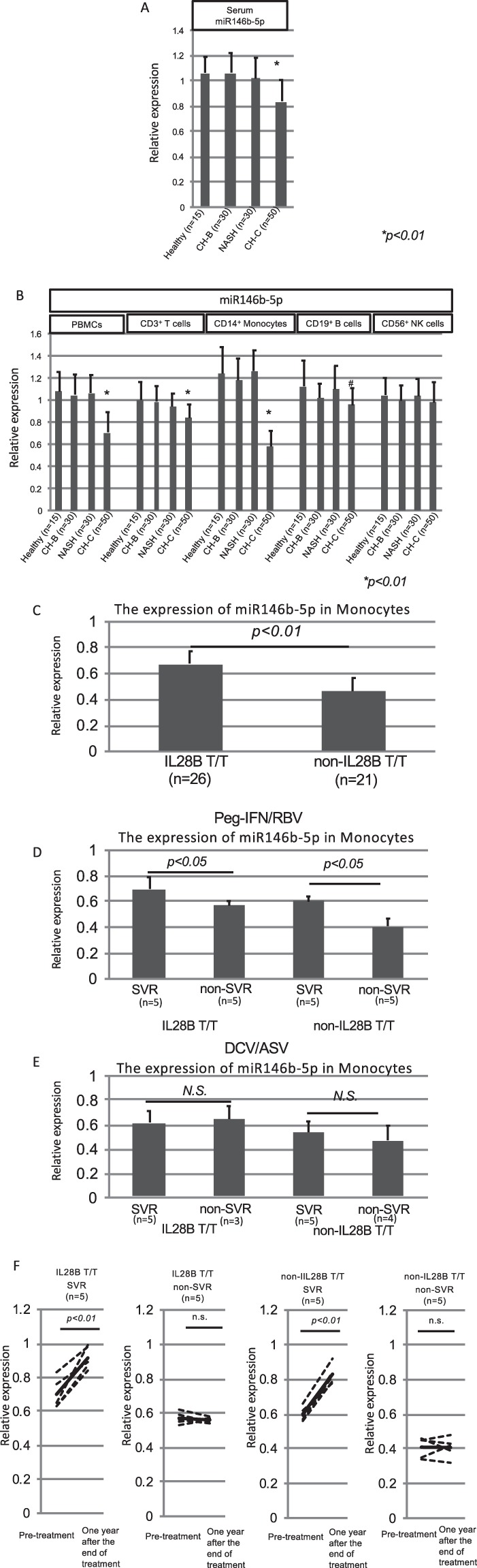
The validation analysis of miR146b-5p expression and the identification of responsive immune cells producing hsa-miR146b-5p. The quantification of miR146b-5p in the serum was carried out to validate the comprehensive analysis using real-time PCR. The Cel-miR-39-3p was spiked in the serum for the control miRNA. The relative expressions of miR146b-5p are shown in the Y-axis. One healthy subject indicated one relative expression. We normalized the other subjects using the relative expression (**A**). Then, we analyzed the expression of hsa-miR146b-5p in various kinds of isolated immune cells (PBMCs, CD3^+^ T cells, CD14^+^ monocytes, CD19^+^ B cells and CD56^+^ NK cells). The quantification of miR146b-5p in the various kinds of cells was carried out using real-time PCR. One PBMC sample in a healthy subject indicated one relative expression. Then, we normalized the other samples using the relative expression. The relative expressions of miR146b-5p are shown in the Y-axis (**B**). A comparison of hsa-miR146b-5p expression in monocytes between IL28B T/T (n = 26) and non-IL-28B T/T (n = 21) patients was carried out (**C**). A comparison of hsa-miR146b-5p expression between the SVR patients (n = 10) and non-SVR patients (n = 10) after receiving PEG-IFN/RBV treatment was carried out (**D**). A comparison of hsa-miR146b-5p expression between the SVR patients (n = 10) and non-SVR patients (n = 7) after receiving DCV/ASV treatment was carried out (**E**). Error-bars indicate standard deviation. The expression levels of hsa-miR146b-5p in CD14^+^ monocytes were compared between before and after achieving SVR. (**F**) The relative expressions of miR146b-5p are shown in the Y-axis.

**Figure 4:**
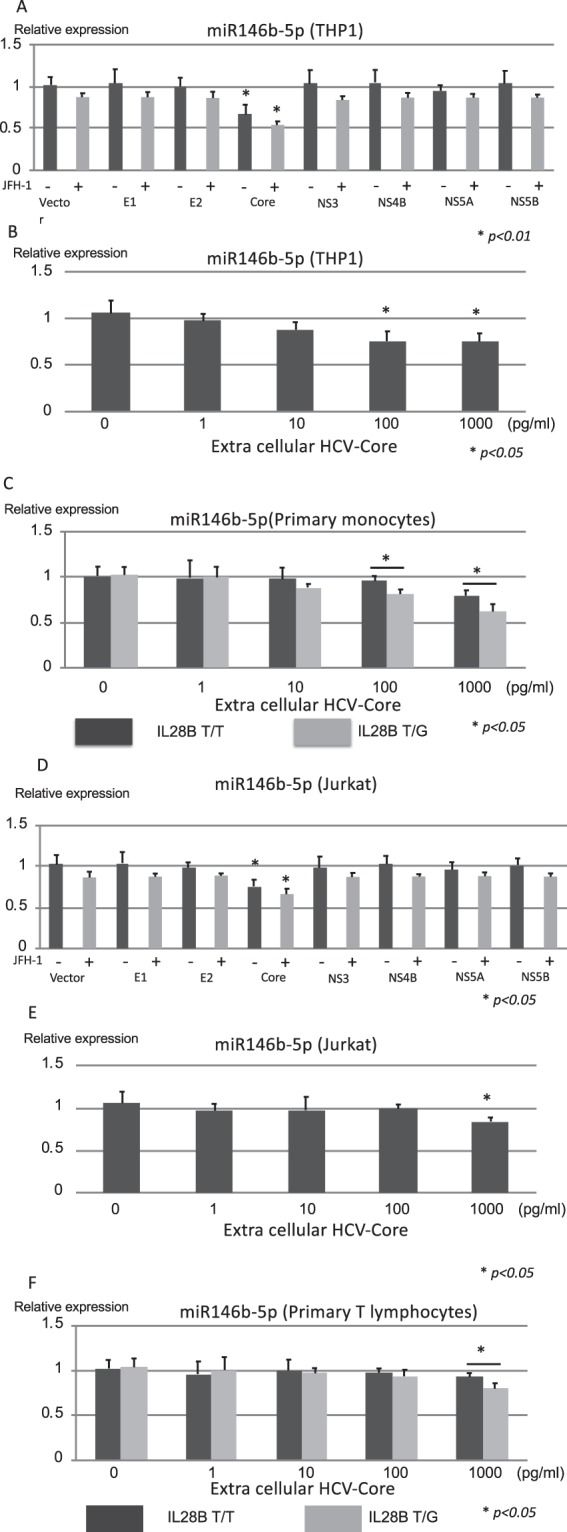
The HCV-antigen responsible for suppressing the expression of hsa-miR146b-5p in monocytes and T cells. The relative expression of miR146b-5p in THP-1 (**A**) and Jurkat (**D**) cells is shown after the transfection of various kinds of HCV antigen expressing plasmids (HCV-core, E1, E2, NS3, NS4B, NS5A and NS5B) with or without JFH-1 full length strain. The relative expressions of miR146b-5p in THP-1 (**B**) and Jurkat (**E**) cells are shown after adding the extra-cellular HCV-core protein. The relative expressions of hsa-miR146b-5p in CD14^+^ monocytes (**C)** and CD3^+^ T cells (**F**) from IL28B T/T subjects and IL28B T/G subjects were analyzed after adding the extra-cellular HCV-core protein. Error-bars indicate standard deviation.

**Figure 5:**
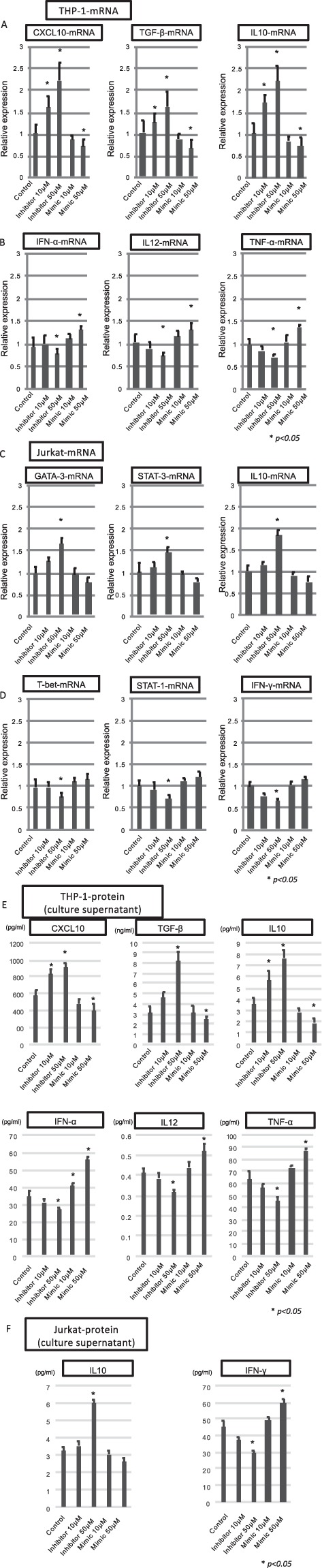
The biological effects of hsa-miR146b-5p in monocytes and T cells. CXCL10, TGF-β and IL10 produced from CD14^+^ monocytes were representative cytokines that could induce favorable effects for eradicating HCV. The expressions of CXCL10-mRNA, TGF-β-mRNA and IL10-mRNA in THP-1 cells are shown after the transfection of the inhibitor or mimic of miR146b-5p (**A**). IFN-α, IL12, and TNF-α produced from CD14^+^ monocytes were representative cytokines that could induce a favorable effect to eradicate HCV. The expressions of IFN-α, IL12, and TNF-α in THP-1 cells are shown after the transfection of the inhibitor or mimic of miR146b-5p (**B**). GATA-3-mRNA, STAT-3-mRNA and IL10-mRNA expressed in T cells were representative factors that could induce unfavorable effects for eradication of HCV. The expressions of GATA-3-mRNA, STAT-3-mRNA and IL10-mRNA in Jurkat cells are shown after the transfection of inhibitor or mimic of miR146b-5p (**C**). T-bet-mRNA, STAT-1-mRNA and IFN-γ-mRNA expressed in T cells were representative factors that could induce favorable effects for eradicating HCV. The expressions of T-bet-mRNA, STAT-1-mRNA and IFN-γ in Jurkat cells are shown after the transfection of inhibitor or mimic of miR146b-5p (**D**). The amounts of CXCL10, TGF-β, IL10, IFN-α, IL12, and TNF-α in the culture supernatant of THP-1 cells are shown after the transfection of the inhibitor or mimic of miR146b-5p (**E**). The amounts of IL10 and IFN-γ in the culture supernatant of Jurkat cells were shown after the transfection of the inhibitor or mimic of miR146b-5p (**F**).Error-bars indicate standard deviation.

**Figure 6:**
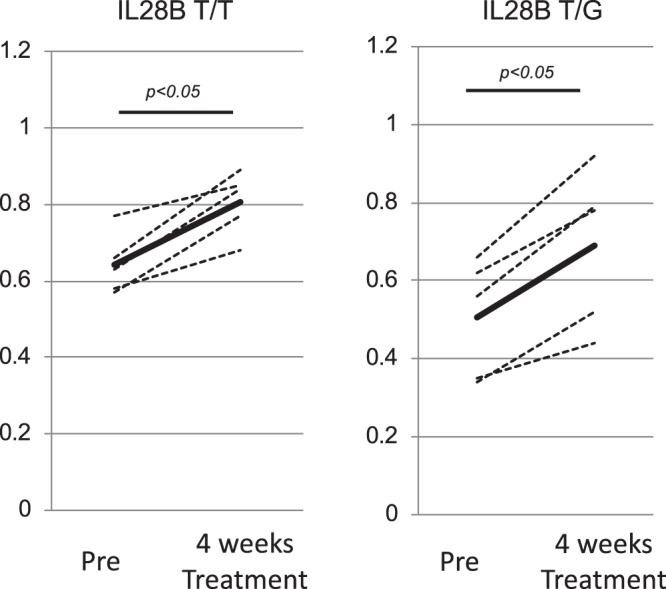
The effect of 1(OH)ViaminD3 treatment on the expression of hsa-miR146b-5p in CH-C patients. The expression of hsa-miR146b-5p in monocytes before and after treatment with 1(OH)Vitamin D3 is shown. The relative expression of hsa-miR146b-5p in monocytes is shown on the Y-axis. One healthy subject indicated one relative expression. Then, we normalized the other subjects using the relative expression.

**Figure 7:**
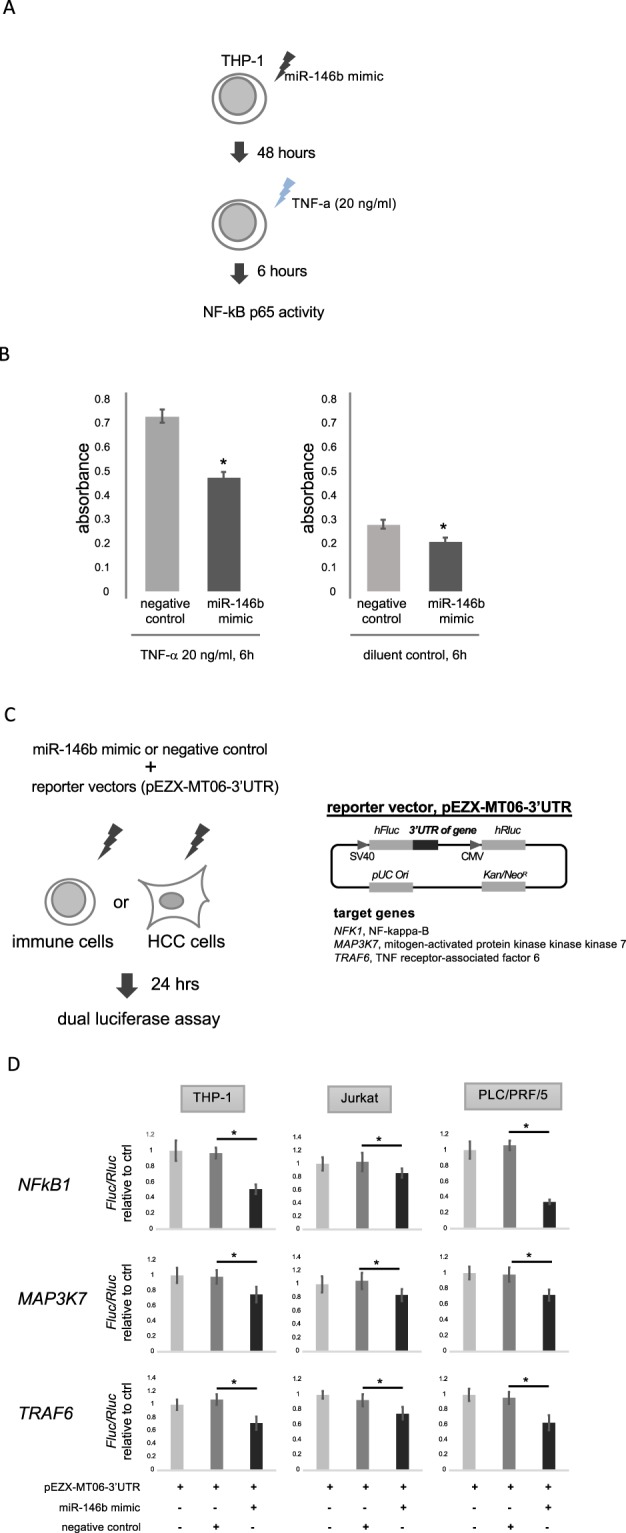
NF-kB transcription assay and reporter assay. THP-1 cells were electroporated with 300 nM of miR-146b mimic or negative control (Ambion) and treated with TNF-α (20 ng/ml) or diluent control for 6 hours (**A**). Nuclear extracts from the cells were prepared using a TransAM Nuclear Extract Kit (Active Motif). The transcription activity of NF-kB was measured using an NF-kB p65 transcription factor assay kit (Active Motif). Bars represent mean absorbance of three determinants ± SE. *p < 0.05 (**B**). THP-1, Jurkat and PLC/PRF/5 cells were electroporated with pEZX-MT06 target reporter vectors containing 3′ UTR of NFKB1, MAP3K7 and TRAF6 (GeneCopoeia) together with miR-146b mimic or negative control (Ambion) (**C**). After 24 hours, luciferase assay was performed using Luc-Pair Luciferase Assay Kit 2.0 (GeneCopoeia). Luminescence of the firefly luciferase was normalized using the Renilla luciferase. Relative luminescence of a reporter vector co-transfected with miR-146b mimic was calculated as a ratio for negative control. Bars represent mean relative luminescence of five determinants ± SE. *p < 0.05 (**D**).

The data in Figs 3A,B, 4A,B,D,E and 5A–F were analyzed by Turkey-Kramer test. The data in Figs 3C,D,E, 4C,F, 6B and 7 were analyzed by independent student *t* test. The data in Figs 3F, 5E were analyzed by paired *t* test. All statistical analyses were carried out using JMP Pro version 9.

## Results

### The quantification and profiles of miRNAs in serum analyzed by deep sequencing

The miRNAs in the serum could be detected in 10 CH-C patients, 10 CH-B patients, and 10 healthy controls (Fig. 1). We selected about 8,000,000 reads per sample for analysis after trimming the reads to exclude ambiguous nucleotides by using cutadapt. The average mapping rates to miRBase and hg19 in CH-C patients were 20.91% and 86.03%, respectively. The differential expression of the miRNA count data was normalized using the trimmed mean of the M values (TMM) normalization process. We standardized the number of individual miRNA reads by the total numbers of 1,000,000 reads in each sample. Then, we selected the differential expression levels of miRNAs by ANOVA. The multiple comparison procedure was applied to compare more than one pair of means; p-values from multiple comparisons were calculated using the false discovery rate (FDR) < 0.1. The expression levels of 21 miRNAs were differentially expressed in the three groups. Among 21 miRNAs, 5 (hsa-miR181a-2-3p, hsa-miR-374a-3p, hsa-miR374a-5p, hsa-miR-204-5p and hsa-miR146b-5p) were shown to be significantly downregulated in CH-C by deep sequence analysis (Fig. 2).

### Comparison between the amounts of serum miRNAs and PBMCs miRNAs

We tried to detect the HCV-specific immune-related miRNAs by comparing the amounts of serum miRNAs and PBMCs miRNA. We selected 4 CH-C patients to isolate serum miRNAs and PBMC miRNAs at the same time. The deep sequence analysis was carried out to quantify both types of miRNAs. We could detect various miRNAs that were highly expressed in PBMCs by analyzing the ratio between the amount of serum miRNAs and PBMC miRNAs (Supplemental Fig. 1, Supplemental Tables 1, 2). The average ratio (PBMCs miRNAs/serum miRNAs) of hsa-miR146b-5p was highest among all miRNAs. Moreover, serum hsa-miR146b-5p was significantly down regulated in CH-C patients in comparison to CH-B patients and healthy subjects.

### Validation study of hsa-miR146b-5p quantification and the identification of responsive immune cells suppressing hsa-miR146b-5p in CH-C patients

Fifty CH-C patients, 30 CH-B patients, 30 NASH patients and 15 healthy individuals were enrolled in the validation study of hsa-miR146b-5p quantification and the identification of responsive immune cells suppressing hsa-miR146b-5p in CH-C. The amounts of hsa-miR146b-5p in the serum and PBMCs of CH-C patients were significantly lower than those in the serum of the other groups (CH-B, NASH and healthy subjects) (*p* < *0.01*)(Fig. 3A,B). Then, we analyzed the expression of hsa-miR146b-5p in various kinds of isolated immune cells (CD3^+^ T cells, CD14^+^ monocytes, CD19^+^ B cells and CD56^+^ NK cells). The expression of hsa-miR146b-5p in CD3^+^ T cells and CD14^+^ monocytes of the CH-C patients was significantly lower than in the other groups (CH-B patients, NASH patients and healthy subjects) (Fig. 3B)(*p* < *0.05*). Especially, the expression of hsa-miR146b-5p in CD14^+^ monocytes of CH-C patients was remarkably lower than in the other groups (CH-B patients, NASH patients and healthy subjects)(Fig. 3B) (*p* < *0.01*).

### IL28B polymorphism and treatment response affect the expression levels of hsa-miR146b-5p in CD14^+^ monocytes

We tried to compare the expression levels of hsa-miR146b-5p in CD14^+^ monocytes between IL28B (rs8099917) T/T and non-IL28B T/T (T/G or GG). The expression of hsa-miR146b-5p in CD14^+^ monocytes of IL28B T/T patients was significantly higher than in those of non-IL28B T/T patients (Fig. 3C). Moreover, a comparison of miR146b-5p expression between the sustained virological response (SVR) patients and non-SVR patients after pegirated-interferon/ribavirin therapy and dacratasvir (DCV) and asunaprevir (ASV) therapy was carried out. The hsa-miR146b-5p expression in CD14^+^ monocytes of the SVR patients treated with Peg-IFN/RBV was significantly higher than in those of the non-SVR patients treated with Peg IFN/RBV(Fig. 3D). However, the hsa-miR146b-5p expression in CD14^+^ monocytes of the SVR patients treated with DCV and ASV was comparable to that of the non-SVR patients treated with DCV and ASV(Fig. 3E). Moreover, the expression levels of hsa-miR146b-5p in CD14^+^ monocytes were significantly increased after achieving SVR(Fig. 3F).

### The HCV-antigens responsible for suppressing the expression of hsa-miR146b-5p in monocytes and T cells

We tried to detect the HCV antigens that could suppress the expression of hsa-miR146b-5p by using various kinds of HCV antigen-expressing plasmids (HCV-core, E1, E2, NS3, NS4B, NS5A and NS5B). The transfection efficiencies of the various kinds of plasmids were 60–65% in THP-1 cells and Jurkat cells since it was difficult to transfect plasmids into lymphoid cells. The expression of HCV-core protein and/or extra cellular HCV-core protein could significantly suppress hsa-miR146b-5p in THP-1 cells, Jurkat cells, primary monocytes and primary T lymphocytes. (Figs 4A–F). However, the core expressing plasmid was able to reduce miR-146b-5p expression to approximately 30%. The low efficiency of transfection might affect the low reduction of miR-146b-5p. The supernatant of Huh-7 cells transfected with JFH-1 strains at 10 days post-transfection was used to examine whether the full length HCV clone together with individual viral proteins could induce greater inhibition of miR146b-5p in THP-1 and Jurkat cell lines. We observed better inhibition of miR146b-5p in monocytes with HCV-core and full length HCV clone. However, the additive effect of full length HCV clone was limited (Fig. 4A,D). Then, we analyzed the effect of extra-cellular HCV-core protein on the expression of hsa-miR146b-5p in monocytes and T cells (Fig. 4B.E). The extra-cellular HCV-core protein could dose-dependently suppress the expression of hsa-miR146b-5p in THP-1 cells and primary monocytes (Fig. 4B,C). Moreover, the expression of hsa-miR146b-5p in monocytes from IL28B T/G subjects was significantly lower than that in monocytes from IL28B T/T subjects after adding the HCV-core protein (Fig. 4C). On the other hand, only high amounts of extra-cellular HCV-core protein could suppress hsa-miR146b-5p in Jurkat cells and primary monocytes (Fig. 4E,F).

### The biological effects of hsa-miR146b-5p in Monocytes and T cells

We analyzed the biological effects of hsa-miR146b-5p in CD14^+^ monocytes. The mimic or inhibitor of hsa-miR146b-5p was transfected into the THP-1 cell line (monocytes) and Jurkat (T cells). Then, we analyzed the expression of cytokine-mRNAs by real-time PCR. We selected CXCL10, TGF-β and IL10 as the immune-suppressive factors that could be produced by monocytes^25,39^. IFN-α, IL12, and TNF-α were selected for the favorable factors that could enhance the eradication of HCV. The expression of CXCL10-mRNA, TGF-β-mRNA and IL10-mRNA was significantly increased after the inhibition of hsa-miR146b-5p in THP-1 cells (Fig. 5A). On the other hand, the expression of CXCL10-mRNA, TGF-β-mRNA and IL10-mRNA were significantly decreased after the transfection of mimic of hsa-miR146b-5p in THP-1 cells (Fig. 5A). Moreover, the expressions of IFN-α, IL12 and TNF-α were significantly decreased after the transfection of inhibitor of hsa-miR146b-5p in THP-1 cells (Fig. 5B). On the other hand, the expressions of IFN-α, IL12 and TNF-α were significantly increased after the transfection of mimic of hsa-miR146b-5p in THP-1cells (Fig. 5B). GATA-3 is an important factor for the induction of Th2 cells. STAT-3 is a factor that could induce Th17 cells. IL10 was an immune-suppressive cytokine produced from Tregs. These factors could inhibit the induction of Th1 cells directly or indirectly. The expressions of GATA-3-mRNA, STAT-3-mRNA and IL10-mRNA were significantly increased after the inhibition of hsa-miR146b-5p in Jurkat cells (Fig. 5C). T-bet, STAT-1 and IFN-γ are Th1-related factors that could enhance the favorable immune response for the eradication of HCV. On the other hand, the expressions of T-bet-mRNA, STAT-1-mRNA and IFN-γ-mRNA were suppressed after the transfection of inhibitor of hsa-miR146b-5p in Jurkat cells (Fig. 5D). In addition to the change of miRNA expression, the protein amounts of cytokines in the culture supernatant were significantly changed after the transfection of miR146b-5p mimic or inhibitor (Fig. 5E,F). The suppression of miR-146b-5p in the monocytes and T cells might contribute to the persistent infection of HCV by complexed mechanisms. The change of Th1-related factors appeared to be small in this result. However, a small change of Th1-related factors could contribute to the suppression of HCV by affecting various kinds of downstream mechanisms.

### 1(OH)ViaminD3 treatment could increase the expression of hsa-miR146b-5p in CH-C patients

Previously, we reported that 1(OH)vitamin D3 treatment could modify the immunological status and improve the treatment response of Peg-IFN/RBV in CH-C patients^25^. However, the mechanisms of immunological modification by 1(OH)vitamin D3 treatment have not been clarified. The change of cytokines after the expression of miR146b-5p was similar to that after the treatment of1(OH)vitamin D3^25^. Therefore, we analyzed the expression of hsa-miR146b-5p in monocytes before and after treatment of 1(OH)Vitamin D3. The treatment of 1(OH)Vitamin D3 could significantly increase the expression of miR146b-5p in monocytes(*p* < *0.05*) (Fig. 6).

### NF-κB signaling could contribute to the hsa-miR146b-5p related immune-modulation in CH-C patients

The DAVID gene functional classification tool revealed a significant enrichment of several gene ontologies with a p-value less than 0.05. The lists of top 10 gene ontologies are shown in Supplemental Table 4. Six gene ontologies related to the regulation of cell death and apoptosis were found and one gene ontology related to NF-κB signaling was identified in the biological function category. Four gene ontologies related to vesicles were identified in the cellular component category. Interestingly, network analysis of the target genes of miR-146b using the String v10 program indicated the central involvement of nuclear factor-kappa B (NF-κB) signaling (Supplemental Fig. 2). Taken together, the analysis predicted that NF-κB signaling is the most likely candidate that would be modulated by miR-146b in the immune response to HCV infection(Fig. 7A,B). A direct interaction between miR-146b and mRNAs related to NF-kB signaling was identified by network analysis. We conducted a reporter assay using miRNA 3′ UTR target clones. 24 hours after co-transfection of miR-146b mimic and 3′UTR of NFKB1, MAP3K7 and TRAF6, the luciferase activity significantly decreased compared to the negative control in two immune cell lines and an HCC cell line (Fig. 7C,D), which indicated that miR-146b directly regulates NF-kB signaling.miR-146b was mainly expressed in the immune cells. HCV core protein suppressed the expression of miR146b-5p in the immune cells. miR146b-5p could suppress NF-κB activity. Therefore, the expression of HCV core in the monocytes could enhance the NF-κB activity. However, we reported that HCV-core protein itself could suppress several types of cytokine signaling such as STAT-1. Therefore, HCV-core protein with NF-κB enhancement might induce various kinds of immunological responses.

## Discussion

The profiling of miRNA expression in serum could be affected by the expression of miRNAs from multiple organs. However, analysis of the serum miRNAs might have some benefits compared to the analysis of those of specific organs. One of the benefits is that the profiling of miRNA expression in the serum must be affected by the whole effects of specific diseases^40,41^. The other benefit is that for the analysis of serum miRNAs and soluble factors it would be relatively easier to collect samples compared to samples from specific organs^35^. Therefore, we used serum samples to identify the CH-C specific miRNA. The expression of five miRNAs was significantly lower in the serum from CH-C patients in comparison to that from CH-B and Healthy subjects. Moreover, the mi146b-5p was abundantly expressed in PBMCs. Other groups also reported that immune-related miRNAs could affect various kinds of immunological functions^42–45^. We carried out a validation analysis to confirm the down-regulation of miR146b-5p in the CH-C patients. Among the PBMCs, the expression of miR146b-5p was remarkably suppressed in CD14^+^ monocytes and CD3^+^ T cells. Moreover, the expression of miR146b-5p could affect the immunological functions of CD14^+^ monocytes and CD3^+^ T cells. In our study, the high expression of miR146b-5p could induce a favorable immune response for the treatment of IFN-based therapy. Moreover, the extracellular HCV-core antigen might be a responsible antigen that could reduce the expression of miR146b-5p, especially in IL28B non-TT subjects. Previously, our group reported that various kinds of HCV antigen could affect the biological functions of immune cells^12,18–20,23,46^. The results of gene ontology analysis indicated that NFκB signaling could be affected by miR146b-5p. miR-146b was mainly expressed in the immune cells. Therefore, the direct effect of HCV replication might not be important. However, the indirect effects by immune cells could affect the HCV replication by changing the miR146b-5p expression. The signaling of NFκB could induce different immune responses according to the basal immune levels and cell types. Moreover, we reported that HCV-core protein itself could suppress several cytokines signaling such as STAT-1. Therefore, HCV-core protein with NF-κB enhancement might induce various kinds of immunological responses. Previously, we reported that 1(OH)Vitamin D3 treatment could induce a favorable immune response in CH-C patients^25^. In this study, the treatment of 1(OH)Vitamin D3 could recover the expression of hsa-miR146b-5p in the monocytes from CH-C patients. Therefore, the favorable immune response during 1(OH)Vitamin D3 treatment could be induced by the up-regulation of hsa-miR146b-5p. The mechanisms of immune modulation by vitamin D3 are complex. Moreover, we need to consider the basal immune response of diseases since the responses to 1(OH)Vitamin D3 differed between IL28B TT subjects and IL28B non-TT subjects.

In conclusion, by our comprehensive analysis we could find specific-miRNAs that were suppressed in PBMCs from CH-C patients. Moreover, hsa-miR146-5p could contribute to the immunopathogenesis of CH-C persistent infection by modifying the cytokine expression. The expression levels of hsa-miR146b-5p could affect the treatment response to Peg-IFN/RBV therapy but not the treatment response to DAA treatment without Peg-IFN. The addition of 1(OH)vitamin D3 might improve the treatment response of Peg-IFN based treatment by up-regulating hsa-miR146b-5p. These findings prove that specific miRNA expressions affect the immune reactions in CH-C patients.

## Supplementary information

Supplemental materials
