# Supplementary material for: The reduction of miR146b-5p in monocytes and T cells could contribute to the immunopathogenesis of hepatitis C virus infection
Source: Sci Rep. 2019 Sep 16;9:13393. doi: 10.1038/s41598-019-49706-9 (PMC6746729; doi:10.1038/s41598-019-49706-9)
Supplement: Supplementary file 1 — Supplemental materials [file 41598_2019_49706_MOESM1_ESM.pdf]

Supplemental materials

The reduction of miR146b-5p in monocytes and T cells could contribute to the immunopathogenesis of hepatitis C virus infection

Yasuteru Kondo[1,4]*, Takayuki Kogure[2], Masashi Ninomiya[2], Ryo Fukuda[1], Norikazu Monma[3], Kazuho Ikeo[3], Yasuhito Tanaka[4]

1. Department of Hepatology, Sendai Kousei Hospital
4-15 Hirose, Aoba, Sendai City, Miyagi, Japan
2. Division of Gastroenterology, Tohoku University Hospital
1-1 Seiryo, Aoba, Sendai City, Miyagi, Japan
3.Center for information Biology, National Institute of Genetics, Mishima, Japan
4. Department of Virology & Liver unit
Nagoya City University Graduate School of Medical Sciences,
Kawasumi, Mizuho, Nagoya 467-8601, Japan.

Corresponding Author
Yasuteru Kondo MD/PhD
Chief Director
Department of Hepatology
Sendai Kousei Hospital
Telephone: +81-22-222-6181, E-mail: yasuteru@ebony.plala.or.jp

Supplemental Figures legend

Supplemental Figure 1

We selected 4 CH-C patients to isolate serum miRNAs and PBMCs miRNAs at the same time. The deep sequence analysis was carried out to quantify both miRNAs. We could detect various miRNAs that were highly expressed in PBMCs by analyzing the ratio between the amounts of serum miRNAs and PBMCs miRNAs. The Y-axis shows the expression of miRNAs in the serum. X-axis shows the expression of miRNAs in PBMCs.

Supplemental Figure 2

To identify the potential roles of miR-146b, protein-to-protein interactions of the target genes of miR-146b were analyzed. DIANA-TarBase v7.0[1], a database that indexes experimentally validated microRNA targets, was used to identify the target genes of miR146b. The list of target genes is shown in supplemental table 3. The gene functional classification tool from the Database for Annotation, Visualization and Integrated

Discovery (DAVID) was used to identify enriched gene ontology (GO) terms in the target genes of miR-146b[2]. The protein-to-protein networks of the target genes were analyzed using the String v10 program[3]. The DAVID gene functional classification tool revealed a significant enrichment of several gene ontologies with a *p*-value of less than 0.05. The lists of top 10 gene ontologies are shown in supplemental table 4. Six gene ontologies related to the regulation of cell death and apoptosis were found and one gene ontology related to NF-kB signaling was identified in the biological function category. Four gene ontologies related to vesicles were identified in the cellular component category. Interestingly, network analysis of the target genes of miR-146b using the String v10 program indicated the central involvement of nuclear factor-kappa B (NF-kB) signaling (supplemental figure 2). Taken together, the analyses predicted NF-kB signaling as the most likely candidate that would be modulated by miR-146b in the immune response in HCV infection.

Reference

1. Vlachos IS, Paraskevopoulou MD, Karagkouni D, Georgakilas G, Vergoulis T, Kanellos I, Anastasopoulos IL, Maniou S, Karathanou K, Kalfakakou D, Fevgas A, Dalamagas T, Hatzigeorgiou AG. DIANA-TarBase v7.0: indexing more than half a million experimentally supported miRNA:mRNA interactions. Nucleic Acids Res. 2015 Jan;43(Database issue):D153-9. doi: 10.1093/nar/gku1215.
2. Huang DW, Sherman BT, Lempicki RA. Systematic and integrative analysis of large gene lists using DAVID Bioinformatics Resources. Nature Protoc. 2009;4(1):44-57.
3. Szklarczyk D, Franceschini A, Wyder S, Forslund K, Heller D, Huerta-Cepas J, Simonovic M, Roth A, Santos A, Tsafou KP, Kuhn M, Bork P, Jensen LJ, von Mering C. STRING v10: protein-protein interaction networks, integrated over the tree of life. Nucleic Acids Res. 2015 Jan; 43:D447-52.

Supplemental Figure 1

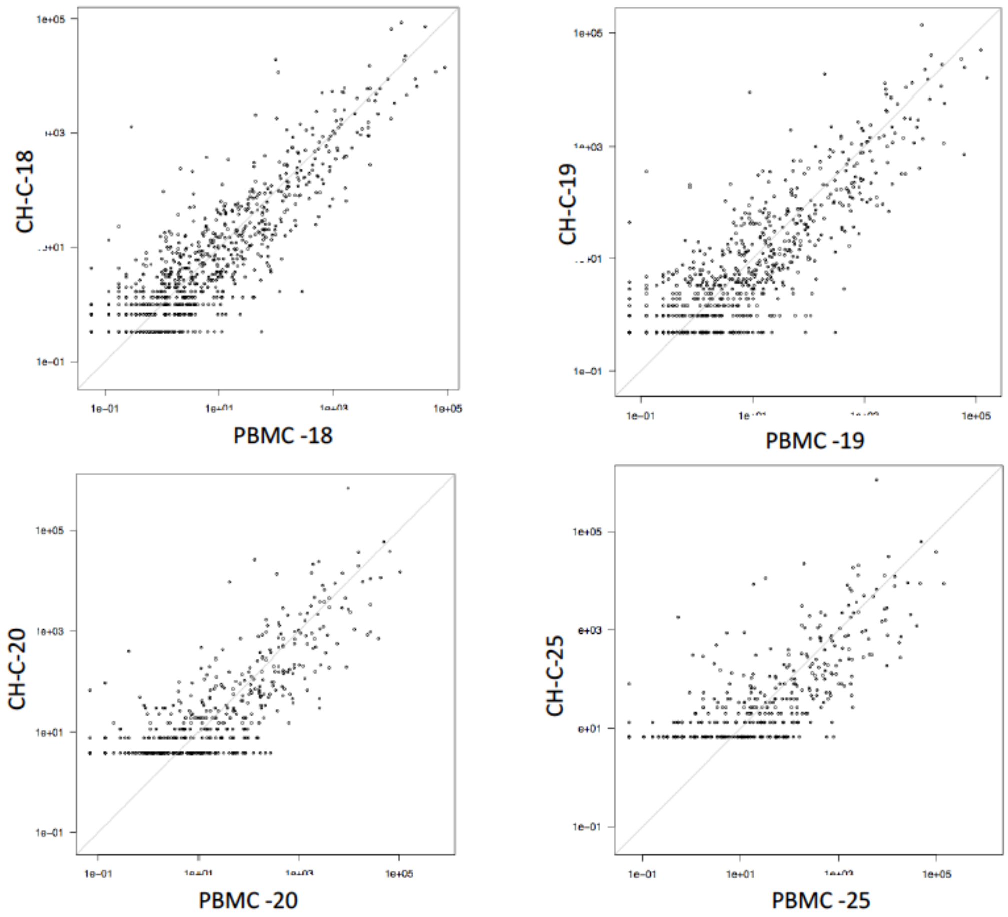

We selected 4 CH-C patients to isolate serum miRNAs and PBMCs miRNAs at the same time. The deep sequence analysis was carried out to quantify both miRNAs. We could detect various miRNAs that were highly expressed in PBMCs by analyzing the ratio between the amounts of serum miRNAs and PBMC miRNAs.

Supplemental Table 1. The various miRNAs that were highly expressed in PBMCs.

ID	*PBMC-1	CH-C-18	ratio	*PBMC-2	CH-C-19	ratio	*PBMC-3	CH-C-20	ratio	*PBMC-4	CH-C-25	ratio
hsa-miR-146b-5p	1662.94	64.57	**25.75**	3094.33	213.20	**14.51**	3967.97	190.91	**20.78**	9631.28	186.72	**51.58**
hsa-miR-664-3p	487.14	24.63	**19.78**	445.82	30.12	**14.80**	730.60	45.82	**15.95**	557.34	53.35	**10.45**
hsa-miR-155-5p	865.91	52.26	**16.57**	2718.33	69.31	**39.22**	2567.45	30.55	**84.05**	1929.47	26.67	**72.34**
hsa-miR-342-3p	4359.98	276.27	**15.78**	9621.39	405.85	**23.71**	8781.33	194.73	**45.09**	18243.76	273.41	**66.73**
hsa-miR-142-3p	1602.08	108.51	**14.76**	2122.74	93.69	**22.66**	2562.18	45.82	**55.92**	1826.84	46.68	**39.14**
hsa-miR-500a-3p	967.02	70.23	**13.77**	1684.80	87.48	**19.26**	470.36	53.46	**8.80**	1312.35	73.35	**17.89**
hsa-miR-3153	71.78	6.99	**10.27**	62.25	10.52	**5.92**	90.54	11.45	**7.90**	111.76	20.01	**5.59**
hsa-miR-140-5p	70.50	9.99	**7.06**	51.30	2.87	**17.89**	74.51	3.82	**19.51**	73.66	6.67	**11.05**
hsa-miR-181a-3p	575.30	90.87	**6.33**	563.94	70.75	**7.97**	646.71	57.27	**11.29**	606.31	40.01	**15.15**
hsa-miR-142-5p	10153.68	1769.44	**5.74**	25949.71	1142.01	**22.72**	23380.71	817.11	**28.61**	19023.94	726.87	**26.17**
hsa-miR-4286	493.76	90.54	**5.45**	396.90	35.85	**11.07**	365.64	26.73	**13.68**	783.77	6.67	**117.53**
hsa-miR-181a-5p	61439.96	11723.36	**5.24**	153745.57	15749.56	**9.76**	105101.30	14734.73	**7.13**	140248.21	8789.08	**15.96**

The various miRNAs that were highly expressed in PBMCs by analyzing the ratio between the amount serum miRNAs and PBMCs miRNAs .

Supplemental Table 2. The various miRNAs that were highly expressed in serum.

ID	*PBMC-1	CH-C-18	ratio	*PBMC-2	CH-C-19	ratio	*PBMC-3	CH-C-20	ratio	*PBMC-4	CH-C-25	ratio
hsa-miR-122-5p	0.00	1171.64	0.00	0.00	1242.87	0.00	0.00	1057.66	0.00	0.00	4274.51	0.00
hsa-miR-3591-3p	0.00	283.92	0.00	0.13	352.78	0.00	0.00	320.74	0.00	0.00	920.25	0.00
hsa-miR-3591-5p	0.00	7.32	0.00	0.00	2.39	0.00	0.00	3.82	0.00	0.00	13.34	0.00
hsa-miR-4732-3p	0.00	39.28	0.00	0.06	44.46	0.00	0.14	95.46	0.00	0.05	80.02	0.00
hsa-miR-483-3p	0.00	28.29	0.00	0.38	5.26	0.07	0.89	30.55	0.03	0.00	46.68	0.00
hsa-miR-375	0.29	1285.14	0.00	0.75	205.07	0.00	0.41	381.83	0.00	0.53	1840.51	0.00
hsa-miR-451a	96.81	19227.50	0.01	8.88	8634.61	0.00	352.84	13398.33	0.03	32.61	11363.12	0.00
hsa-miR-483-5p	0.17	22.97	0.01	0.00	33.94	0.00	0.07	68.73	0.00	0.00	26.67	0.00
hsa-miR-4732-5p	0.12	13.31	0.01	0.00	8.13	0.00	0.21	15.27	0.01	0.05	13.34	0.00
hsa-miR-144-3p	2.09	238.32	0.01	0.75	184.52	0.00	10.75	187.10	0.06	1.06	306.75	0.00
hsa-miR-10b-5p	107.38	11406.16	0.01	193.63	18668.88	0.01	128.82	25937.55	0.00	194.66	22412.83	0.01
hsa-miR-885-5p	1.05	95.20	0.01	1.13	13.86	0.08	0.68	49.64	0.01	1.69	293.41	0.01
hsa-miR-215	3.37	213.02	0.02	6.38	53.06	0.12	7.60	95.46	0.08	6.02	80.02	0.08
hsa-miR-144-5p	5.98	373.79	0.02	4.00	213.20	0.02	29.17	416.19	0.07	3.22	493.47	0.01
hsa-miR-486-3p	42.86	2015.08	0.02	48.05	1964.21	0.02	40.06	9316.59	0.00	18.36	8489.00	0.00
hsa-miR-183-5p	3.54	86.21	0.04	3.25	105.64	0.03	3.97	64.91	0.06	2.90	206.72	0.01
hsa-miR-100-5p	14.34	339.18	0.04	9.70	119.03	0.08	21.30	282.55	0.08	12.03	893.58	0.01
hsa-miR-30a-5p	100.12	1782.75	0.06	135.20	990.95	0.14	233.06	1260.03	0.18	255.19	1927.20	0.13
hsa-miR-145-3p	1.10	17.97	0.06	1.06	19.60	0.05	1.44	30.55	0.05	1.58	13.34	0.12
hsa-miR-125b-1-3p	0.46	4.99	0.09	0.06	3.82	0.02	1.10	11.45	0.10	0.47	13.34	0.04
hsa-miR-4507	0.64	4.99	0.13	0.44	5.74	0.08	0.34	3.82	0.09	0.63	6.67	0.09
hsa-miR-4486	0.17	1.33	0.13	0.94	5.26	0.18	0.00	3.82	0.00	0.16	6.67	0.02
hsa-miR-182-5p	150.53	1143.34	0.13	256.51	2246.73	0.11	218.61	1626.59	0.13	178.15	3100.85	0.06
hsa-miR-485-3p	40.36	299.90	0.13	15.08	360.91	0.04	10.82	458.19	0.02	5.28	880.24	0.01
hsa-miR-3130-3p	0.46	3.33	0.14	0.69	3.82	0.18	0.27	11.45	0.02	0.16	13.34	0.01
hsa-miR-654-3p	62.90	397.42	0.16	20.58	349.44	0.06	7.12	53.46	0.13	5.33	53.35	0.10
hsa-miR-5010-5p	2.50	15.64	0.16	4.69	75.53	0.06	2.47	15.27	0.16	1.69	40.01	0.04
hsa-miR-4432	1.97	11.98	0.16	0.63	21.03	0.03	0.82	34.36	0.02	0.95	13.34	0.07
hsa-miR-486-5p	15274.34	85763.18	0.18	10648.73	137545.17	0.08	9490.43	687950.26	0.01	5973.69	1140300.22	0.01

The various miRNAs that were highly expressed in serum by analyzing the ratio between the amounts of serum miRNAs and PBMCs miRNAs

Supplemental Figure 2

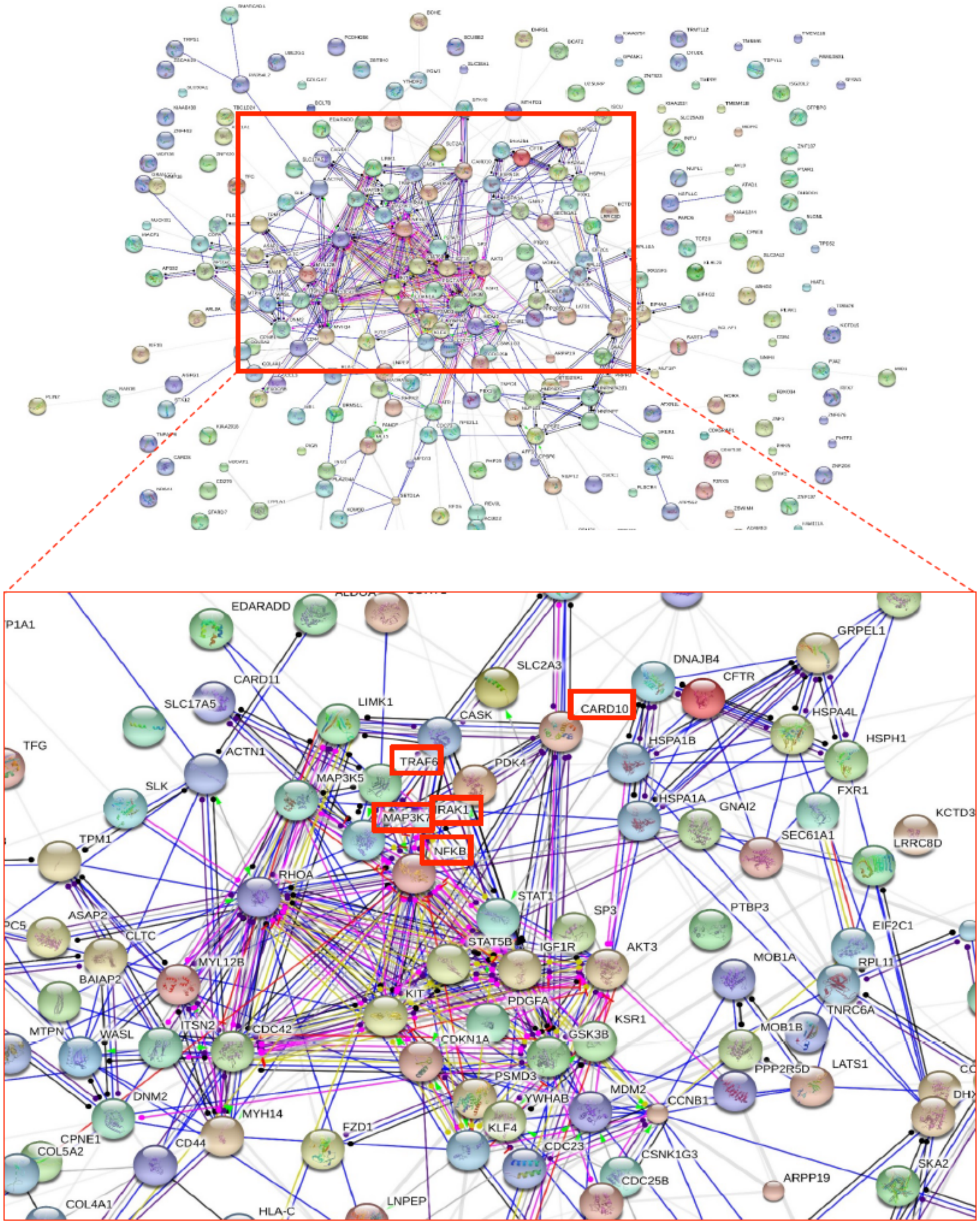

Supplemental Table 3. Target genes of miR14b-5p (DIANA-TarBase v7.0)

Gene symbol	Gene ID	Gene symbol	Gene ID	Gene symbol	Gene ID	Gene symbol	Gene ID	Gene symbol	Gene ID
PEAK1	79834	ACTN1	87	PPP2R5D	5528	CARD8	22900	NSFL1C	55968
U2SURP	23350	CFTR	1080	MTHFD1	4522	ARPP19	10776	CARD11	84433
PHF20L1	51105	MTPN	136319	UBN2	254048	ZNF597	146434	SESN3	143686
CLTC	1213	BCL7B	9275	ABRACL	58527	GPANK1	7918	HIAT1	64645
LIMK1	3984	LNPEP	4012	MAP3K5	4217	NR6A1	2649	BCLAF1	9774
RAB18	22931	GINS1	9837	EIF4G2	1982	ING3	54556	KIAA1244	57221
PLA1A	51365	CD276	80381	TMBIM6	7009	CDC25B	994	PJA2	9867
STIM1	6786	SPEN	23013	MDM2	4193	EIF4A3	9775	AKT3	10000
TRMT112	51504	ABHD17B	51104	ZC3H10	84872	MEA1	4201	CDC73	79577
RPL11	6135	C1RL	51279	MAML1	9794	BCAT2	587	TXLNG	55787
TNFAIP8	25816	LRRC36	55282	STK40	83931	LTA4H	4048	HSPA4L	22824
TMEM216	51259	PHTF2	57157	SLC25A33	84275	WASL	8976	ZNF823	55552
BLOC1S2	282991	GOLGA7	51125	CPNE8	144402	TNPO1	3842	RORA	4919
PLS3	5358	SLC2A3	6515	NUPL1	9818	ETAA1	54465	SREK1	140890
ATP1A1	476	ITSN2	50618	MAP3K7	6885	BRMS1L	25855	DHX8	1659
SART3	9733	FAM160A1	729830	DCAF16	54876	OTUD1	220213	CDK5RAP1	51654
BAIAP2	10458	ABHD2	11057	LATS1	9113	GSK3B	2932	WDR36	134430
CCL3	6348	ZNF107	51427	DNAJB4	11080	SLC38A1	6303	TRAF6	7189
ATR	545	STAT5B	6777	FAM111A	63901	EPB41L4A	64097	SLC19A2	10560
CSDE1	7812	IGF1R	3480	PTAR1	375743	SLK	2534	FBXO28	23219
CD84	8832	AGFG1	3267	NUPL2	11097	Scube2	57758	AGO1	26523
MACF1	23499	MOB1B	92597	ZNF367	195828	HSPA1A	3303	YTHDF2	51441
PAPD5	64282	SP3	6670	STAT1	6772	TMPPE	643853	ZNF676	163223
PSMD3	5709	KIAA0430	9665	COPA	1314	MMP16	4325	SOGA1	140710
ASAP2	8853	CPSF6	11052	OGFOD1	55239	NFKB1	4790	FBXO34	55030
PDGFA	5154	PBX1	5087	ZNF92	168374	MYL12B	103910	ICE1	23379
CD44	960	PPRC1	23082	AP3S2	10239	ZNF197	10168	KBTBD6	89890
LYPLA1	10434	EXOC6B	23233	GRPEL1	80273	PLA2G4A	5321	MID1	4281
KLF4	9314	CPSF2	53981	CDC23	8697	CASK	8573	HSPH1	10808
SETD1A	9739	SORT1	4877	FANCF	2188	ZNF43	7594	SNRNP27	11017
ATP5G2	517	KIF1B	23095	JADE2	23338	ATAD1	84896	YWHAB	7529
USP33	23032	CPNE1	1369	PIGB	9488	BCL2L13	23786	KIAA2018	205717
PHKB	5257	DNM2	1785	NLGN1	22871	DNPH1	10591	ZSCAN29	146050
DDX26B	203522	RFX5	5993	IQCE	23288	POMT2	29954	RHPN2	85415
ATP13A3	79572	NUCKS1	64710	FXR1	8087	ZNF3	7551	IER2	9592
IRAK1	3654	KSR1	8844	TCF20	6942	ISCU	23479	KLHL20	27252
KMT2E	55904	ARL8A	127829	RBM26	64062	CENPU	79682	SERPINB9	5272
SLC2A12	154091	CCNB1	891	RBL1	5542	TFG	10342	TRPS1	7227
RHBDD1	84236	ADAM10	102	DHRS1	115817	KIT	3815	GMFB	2764
KCTD15	79047	KIAA0754	643314	GTPBP3	84705	MDFIC	29969	PGM1	5236
HNRNPA2B1	3181	RASSF5	83593	ELMSAN1	91748	PLIN2	123	P2RX5	5026
PPA1	5464	AFF1	4299	TP53INP1	94241	MR1	3140	CSNK1G3	1456
AVL9	23080	HLA-C	3107	PAFAH2	5051	RHOA	387	HNRNPD	3184
TNRC6A	27327	ALDOA	226	ZNF493	284443	PRPF8	10594	ATXN1L	342371
EDARADD	128178	HNRNPF	3185	TBC1D24	57465	CARD10	29775	PDK4	5166
RAD54L2	23132	INTU	27152	BTG2	7832	DDHD1	80821	MMGT1	93380

Supplemental Table 4. Top 10 gene ontologies identified in the target genes of miR146b-5p

Biological function

Gene ontology term	*p*-value
regulation of programmed cell death	3.69E-07
regulation of cell death	4.02E-07
regulation of apoptosis	8.77E-07
negative regulation of programmed cell death	2.74E-04
negative regulation of cell death	2.86E-04
negative regulation of apoptosis	7.23E-04
activation of NF-kappaB-inducing kinase activity	0.00108989
cellular protein localization	0.001188208
intracellular protein transport	0.001257681
cellular macromolecule localization	0.001271843

Cellular component

Gene ontology term	*p*-value
nuclear lumen	7.19E-05
organelle lumen	4.72E-04
intracellular organelle lumen	5.77E-04
membrane-enclosed lumen	7.10E-04
nucleoplasm	0.001111336
membrane-bounded vesicle	0.00229947
nucleolus	0.00370583
cytoplasmic membrane-bounded vesicle	0.004065685
vesicle	0.005384863
cytoplasmic vesicle	0.007795455

Molecular function

Gene ontology term	*p*-value
cytoskeletal protein binding	0.001553715
nucleotide binding	0.002700479
RNA binding	0.006939658
caspase regulator activity	0.011474242
cation binding	0.018389259
ion binding	0.01878988
carboxylesterase activity	0.020436556
metal ion binding	0.020653403
protein kinase activity	0.021020565
tubulin binding	0.023904927
